# A hydatic cyst of the appendix mimicking a uterine lateral mass: a case report 

**DOI:** 10.1186/s13256-018-1602-6

**Published:** 2018-03-25

**Authors:** Molka Chemlali, Sarah Sghaier, Montassar Ghalleb, Amira Triki, Jamel Ben Hassouna, Khaled Ben Rahal

**Affiliations:** Surgical Oncology Department, Salah Azaiez Institute of Cancer, Tunis, Tunisia

**Keywords:** hydatic cyst, Ovarian mass, Surgery

## Abstract

**Background:**

Hydatic cyst is a zoonotic disease caused by Echinococcus granulosus. It is a public health problem in Tunisia and remains endemic. It occurs in intra-abdominal organs in 10–15% of the cases, particularly in the liver representing the most common affected organ. The aim of the case is to highlight the scarcity of this appendicle origin and to consider it among the differential diagnosis of any intra-abdominal cystic lesion.

**Case presentation:**

A 45-year-old Tunisian woman, with no past medical history, was admitted for a pelvic cystic mass. The clinical manifestation as well as the imaging findings were toward a lateral uterine mass. Our patient underwent appendectomy and resection of the mass. The patient had no recurrence at 2 years of follow-up.

**Conclusions:**

An extrahepatic hydatid cyst should be recognized among the differential diagnosis of any intra-abdominal cystic lesion. Treatment should be based on surgical excision. Due to the risk of recurrence, a close follow-up is mandatory.

## Background

Hydatidosis is a zoonotic disease caused by the larval stage of tapeworm Echinococcus granulosus [1]. The lifecycle of this parasite includes two hosts: dogs are the definitive ones and humans are the intermediate ones [2]. Humans become accidentally infected by many routes of transmission, including: eating vegetables or drinking water that can both be contaminated with the hydatid ova; and direct contact with the definitive hosts [2]. It is endemic in Tunisia and represents a serious public health problem [3], where the annual human incidence of surgical cases is approximately 15/100,000 [3].

Cysts are mainly found in the liver (63%), succeeded by the lungs (25%), muscles (5%) and bones (5%) [4]. The involvement of the pelvic region is rare and represents an incidence which ranges between 0.2 and 2.25% [4].

We present a case highlighting the importance of considering appendical hydatic cyst among the differential diagnosis when facing a case of intra-abdominal cystic lesion in endemic countries.

## Case presentation

A 45-year-old Tunisian woman, with no past medical history, was admitted for a pelvic mass. She had no associated history of vomiting, fever, jaundice or weight loss. On physical examination, the patient was anicteric and afebrile. The abdominal palpation found a bloating in the right iliac fossa. Her gynecological examination showed a right pelvic mass in the recto-uterine pouch. Her blood tests were normal. The abdominal ultrasound revealed a suspicious cystic mass of 9 cm in diameter, located at the right side of the uterus. The right ovary was not seen. The thoracic-abdominal pelvic scan showed a heterogeneous right lateral rectal mass of 9 cm.

Tumor markers, including CA 125, alpha fetoprotein, Beta human chorionic gonadotropin (hCG) hormone, carcinoembryonic antigen (CEA) and CA19–9 were tested and found within the normal range. With nonspecific signs and symptoms, wide radiological image findings, an anatomical contiguity, and the absence of a clinical history of a previous hepatic echinococcal cyst, a preoperative diagnosis of pelvic tumor was suggested.

Our patient was scheduled for an exploratory laparotomy. Three cystic masses were observed intra-operatively: the first one was 4.5 cm in diameter and was closely located to the appendix; the second one measured 2 cm in diameter and was attached to the uterovesical pouch; and the third cyst was of 6 cm in diameter, and was detected laterally to the right of the rectum. The rest of the abdominal cavity was normal.

An appendectomy and a complete resection of the cysts were conducted. The frozen section showed the presence of hydatic cysts, which were later confirmed by the histopathological examination. The operation ended with peritoneal cleansing with a hypertonic saline solution. Our patient had an uncomplicated postoperative course and was discharged from the hospital on the third postoperative day.

Our patient was then referred to a general surgery department to undergo an appropriate follow-up. We also took the opportunity to integrate her in the breast cancer screening program. Two years after treatment, our patient remained under follow-up and demonstrated no evidence of recurrent hydatidosis.

## Discussion

The liver is the most common affected organ. Other intra-abdominal organs are less concerned and represent only 10–15% of the cases [5]. The involvement of the appendix with this parasitosis is rare even in the endemic areas and it is due to an ectopic migration of the hydatic cyst localized in a different site [1].

The clinical manifestation of the hydatic cyst varies widely and it is associated with nonspecific radiological images, which lead to difficulties in making the correct diagnosis [4], as in the present case. However, our first suspicion was pelvic tumors such as gynecologic tumors, appendicle tumors or mucocele of the appendix.

A hydatic cyst develops increasingly inducing compression and irritation of the adjacent organs, which adds difficulties in diagnosing this pathology [6]. When located in the pelvic region (0.2–2.25%), it may cause abdominal pain and swelling, urinary disorders, or menstruation disturbances [4].

For the abdominal localization, the ultrasonography represents an important imaging examination as it may show the characteristic aspect of this cystic lesion, and may help in making the correct diagnosis [7].

Surgical excision is the preferred treatment [2]. An en bloc resection without rupture is the recommended procedure to avoid spreading of daughter cysts and so the onset of secondary disease [2]. New therapeutic approaches have been described including percutaneous puncture of the cyst called the PAIR procedure (Puncture, Aspiration, Injection, Re-aspiration). Paksoy *et al.* conducted this procedure and showed successful results, considering it safe and simple, but antihelminthic drugs should be administrated postoperatively [8].

The overall local recurrence rate of hepatic disease ranges between 10 and 12% [9], thus a regular follow-up must be undertaken to detect recurrence.

## Conclusions

The involvement of the appendix, especially as a primary localization of the hydatid cyst, is a rare finding. This pathology is associated with nonspecific clinical manifestation and different radiological images. Surgical excision remains the preferred treatment. A close follow-up is highly recommended to detect further recurrence.
